# Malaria distribution and performance of malaria diagnostic methods in Malaysia (1980–2019): a systematic review

**DOI:** 10.1186/s12936-020-03470-8

**Published:** 2020-11-07

**Authors:** Mohd Amirul Fitri A. Rahim, Mohd Bakhtiar Munajat, Zulkarnain Md Idris

**Affiliations:** Department of Parasitology and Medical Entomology, Faculty of Medicine, Universiti Kebangsaan Malaysia, 56000 Kuala Lumpur, Malaysia

**Keywords:** Malaria, Malaysia, Distribution, Diagnostic, Systematic review

## Abstract

**Background:**

Malaysia has already achieved remarkable accomplishments in reaching zero indigenous human malaria cases in 2018. Prompt malaria diagnosis, surveillance and treatment played a key role in the country’s elimination success. Looking at the dynamics of malaria distribution during the last decades might provide important information regarding the potential challenges of such an elimination strategy. This study was performed to gather all data available in term of prevalence or incidence on *Plasmodium* infections in Malaysia over the last four decades.

**Methods:**

A systematic review of the published English literature was conducted to identify malaria distribution from 1980 to June 2019 in Malaysia. Two investigators independently extracted data from PubMed, Scopus, Web of Science and Elsevier databases for original papers.

**Results:**

The review identified 46 epidemiological studies in Malaysia over the 39-year study period, on which sufficient information was available. The majority of studies were conducted in Malaysia Borneo (31/46; 67.4%), followed by Peninsular Malaysia (13/46; 28.3%) and in both areas (2/46; 4.3%). More than half of all studies (28/46; 60.9%) were assessed by both microscopy and PCR. Furthermore, there was a clear trend of decreases of all human malaria species with increasing *Plasmodium knowlesi* incidence rate throughout the year of sampling period. The summary estimates of sensitivity were higher for *P. knowlesi* than other *Plasmodium* species for both microscopy and PCR. Nevertheless, the specificities of summary estimates were similar for microscopy (40–43%), but varied for PCR (2–34%).

**Conclusions:**

This study outlined the epidemiological changes in *Plasmodium* species distribution in Malaysia. Malaria cases shifted from predominantly caused by human malaria parasites to simian malaria parasites, which accounted for the majority of indigenous cases particularly in Malaysia Borneo. Therefore, malaria case notification and prompt malaria diagnosis in regions where health services are limited in Malaysia should be strengthened and reinforced to achieving the final goal of malaria elimination in the country.

## Background

Malaria is one of the most prevalent mosquito-borne infectious diseases in the world. An approximately 228 million malaria cases and 405,000 deaths were reported in 2019 globally [1]. Although an estimated 20 million fewer cases were reported in 2019 than in the previous ten years, no significant progress has been made in reducing global malaria cases over this timeframe [1, 2]. The majority of cases in 2019 were in the World Health Organization (WHO) African Region (213 million or 93%), followed by 3.4% from the WHO South-East Asia Region and the WHO Eastern Mediterranean Region accounted for 2.1% of the overall cases [1]. Of all five species of malaria that infect human i.e. *Plasmodium falciparum*, *Plasmodium vivax*, *Plasmodium malariae*, *Plasmodium ovale* and *Plasmodium knowlesi*. *Plasmodium falciparum* is the most prevalent and causes the highest mortality particularly in the African region [3].

In the WHO Western Pacific Region, there are 753 million people in 10 countries that are currently at risk of infections with malaria [1]. Malaysia, which is included in this region, is in the pre-elimination phase and continues to progress towards elimination, reporting zero indigenous human malaria cases in 2018 [3], which is two years ahead of target elimination in 2020 [4]. This is particularly impressive considering that in 2010, over 5000 cases were reported in the country [3]. Even though malaria control activities have significantly reduced human malaria incidence in Malaysia, the resurgence of the malaria parasite *P. knowlesi* still remains as a main public health problem in the less developed areas of the country, especially in Malaysia Borneo [5–7] and among hard-to-reach populations of indigenous people in Peninsular Malaysia [8–11]. About one-third (32%) of total malaria cases occur in Peninsular Malaysia, and the majority of these are found in the central, south-eastern and northern coastal regions [6]. The remaining 68 percent of cases are found in Malaysian Borneo, primarily the states of Sabah and Sarawak [5]. Previous studies revealed that higher historical forest loss could be one of the factors that were significantly associated with higher incidence of *P. knowlesi* infection in Malaysia [12–16].

Currently, several types of malaria diagnostic methods are available including light microscopy, rapid diagnostic tests (RDTs) and polymerase chain reaction (PCR) assay. In Malaysia, light microscopy examination of blood slides is the primary method in malaria diagnosis [17, 18]. This method remains the gold standard for malaria diagnosis and has clear advantages; it is inexpensive and allows for identification and quantification of malaria species [19, 20]. However, the quality of a diagnosis based on microscopy is often inadequate. The accuracy depends on the level of competence of the microscopist and may be adversely affected by operational limitations or technical problems [19, 21, 22]. Plus, infections with low density are unlikely to be detected by conventional microscopy [23–25]. Unlike microscopy, malaria RDTs requiring no technical equipment and minimal expertise [26]. However, RDTs do not provide parasite quantification and are considered more expensive than light microscopy [19]. Molecular techniques such as PCR are more accurate in identification and differentiation of all malaria species than microscopy and RDT [10, 27–29]. Despite the greater sensitivity of PCR, it is not convenient for field and resource-limited settings due to the requirement of complex equipment, reagents and know-how [19]. The Malaysia government has adopted various strategies to eliminate malaria including access to early diagnosis and treatment, a strong surveillance system and effective vector control measures [17].

Several studies have been conducted to assess prevalence of *Plasmodium* spp. in Malaysia. However, there is no detailed systematic review on malaria epidemiology and information on changes in prevalence or incidence over the past decades. Therefore, the aim of this study was to collate relevant published studies related to the distribution of malaria in Malaysia through a systematic review strategy from 1980 to 2019.

## Methods

### Search strategy

This systematic review was conducted using published studies on the prevalence of malaria in Malaysia. Eligible studies were identified in PubMed, Scopus, Web of Science (Clarivate Analytic) and Elsevier (Science Direct) databases searched from January 1980 to June 2019. The search was commonly conducted using the search term [(“*Plasmodium*” OR “malaria”) AND (“prevalence” OR “epidemiology”) AND “Malaysia”] of combination to obtain relevant articles. This systematic review was accorded to the protocol and followed the PRISMA (Preferred Reporting Items for Systematic Reviews and Meta-analysis) guidelines [30].

### Eligibility criteria

Primary malaria research conducted in Malaysia was included in this study including the previous reports of prevalence or incidence of malaria in the country. Only full-text articles in English were considered. The articles must also provide a description of sample size, study design, study site, malaria diagnosis method as well as duration of study. For exclusion criteria, this study excluded previous articles on case reports, letters, posters, conference abstracts, and studies conducted through experimental works of malaria in animal models. Articles with insufficient data, and literature reviews were also excluded while for cohort studies, data were extracted from the baseline observation only.

### Data extraction

All searched articles were imported into the EndNote X9 version software and then the duplicated files were removed. Based on the predetermined inclusion criteria, two independent review authors (MAFAR and MBM) determined qualified studies based on titles and abstract and from selected articles; the relevant information was extracted in the Microsoft Excel Spreadsheet for analysis. The date extraction sheet included the name of the first author, year of publication, region (state), geographical location (Peninsular or Borneo), study design, study group (subjects), sample size, sampling technique, period of study, diagnostic method (microscopy and/or PCR) and species-specific total positive finding.

### Statistical analysis

Microscopy and/or PCR parasite prevalence were calculated for each study, once for all *Plasmodium* species single infection and mixed infections. Summary descriptive statistics using frequency and percentage for malaria cases were tabulated to obtain a clear understanding of the population studied. Incidence rate (reported per 100,000 population) was calculated based on the number of population in Malaysia for the respective year and region (i.e. sampling area) based on census from the Department of Statistics [31]. Sensitivity and specificity analysis for microscopy and PCR were calculated using microscopy as the reference technique and visually summarized in a box plot for easy-to-read visualization of the test accuracy variance between studies. All analyses were done using STATA SE version 15.1 (Stata Corp, TX, USA).

## Results

### Data and study characteristics

**Fig. 1:**
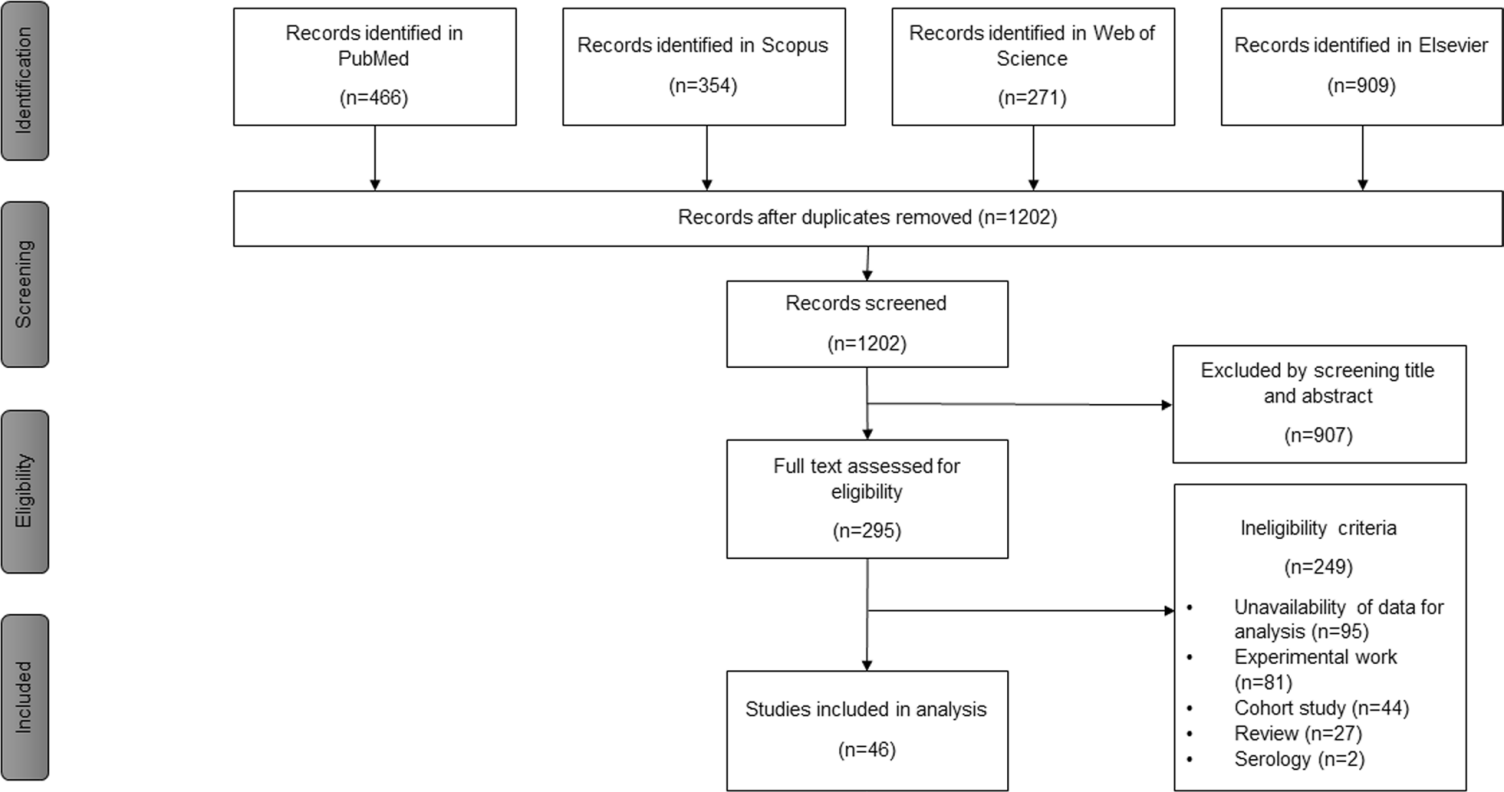
Flowchart of selected articles for the systematic review according to the PRISM statement

The literature search generated 466 results in PubMed, 354 in Scopus, 271 in Web of Science (Clarivate Analytic) and 909 in Elsevier (Science Direct) databases (Fig. 1). After removing duplicates, 1202 articles were left for screening. Following screening of titles and abstracts, 295 studies were retained for more detailed evaluation. The most common reason for exclusion was the unavailability of data for analysis. Other reasons for exclusion including experimental studies, cohort studies, and reviews. As a result, 46 articles were selected in the study for full data extraction [5, 7–9, 23–25, 27, 28, 32–68].

### Description of included studies

Of the 46 included studies, six studies (13%) were published from 1988 to 1999, followed by 11 studies (24%) from 2000 to 2010 and 29 studies (63%) from 2011 to June 2019 (Table 1). The majority of studies were conducted in Malaysia Borneo (31/46; 67.4%), followed by Peninsular Malaysia (13/46; 28.3%) and in both areas (2/46; 4.3%). In term of sampling strategy, 33 studies derived from hospital data, 12 studies from population data and only one study from the combination of both hospital and population data.

**Table 1:** Summary of main features of included articles

No	References	Sample size (N)	Period of sample collection^a^	Study design	Study area^b^	Prevalence by microscopy, n (%)	Pf, Pv, Pm, Po by microscopy, n (%)	Pk by microscopy, n (%)	Mixed infection by microscopy, n (%)	Prevalence by PCR, n (%)	Pf, Pv, Pm, Po by PCR, n (%)	Pk by PCR, n (%)	Mixed infection by PCR, n (%)
1	Jiram et al. [24]	1995	2013–2014	Cross-sectional	Borneo	0 (0)	0 (0)	0 (0)	0 (0)	145 (7.3)	116 (80)	9 (6.2)	20 (13.8)
2	Cooper et al. ^c^ [5]	3867	2015–2017	Hospital	Borneo	3788 (98)	264 (7)	3524 (93)	0 (0)	3580 (99.5)	283 (7.9)	3262 (91.1)	
3	Grigg et al. [32]	852	2012–2016	Hospital	Borneo	846 (99.3)	81 (9.6)		7 (0.8)				
4	Grigg et al. ^c^ [33]	872	2012–2016	Hospital	Borneo	854 (97.9)	309 (36.2)	529 (61.9)		789 (97.3)	300 (38)	482 (61.1)	6 (0.8)
5	Jeffree et al. [34]	470	2012	Cross-sectional	Borneo	11 (2.3)	11 (100)	0 (0)	0 (0)		0 (0)		
6	Liew et al. [35]	3757	2016–2017	Cross-sectional	Peninsular	164 (4.4)	164 (100)	0 (0)	0 (0)	43 (26.5)	43 (100)		
7	Siner et al. [25]	3002	2014–2015	Cross-sectional	Borneo	5 (0.2)	0 (0)	5 (100)	0 (0)	9 (0.3)	1 (11.1)	7 (77.8)	
8	Grigg et al. ^c^ [36]	414	2012–2015	Cross-sectional	Borneo	414 (100)				412 (99.5)	90 (21.8)	268 (65)	2 (0.5)
9	Mohd Abdul Razak et al. ^c^ [37]	4257	2008–2009, 2011, 2014	Cross-sectional	Borneo	112 (2.6)	60 (53.6)			112 (2.6)			
10	Britton et al. ^c^ [38]	261	2012	Hospital	Borneo	149 (57.1)				149 (57.1)	88 (59.1)	56 (37.6)	1 (0.7)
11	Stanis et al. ^c^ [28]	129	2012–2013	Hospital	Borneo	109 (84.5)	101 (92.7)	6 (5.5)	2 (1.8)	103 (79.8)	35 (34)	68 (66)	
12	Fornace et al. 2016^c^ [39]	2006	2008–2012	Hospital	Borneo	1847 (100)	1847 (1847)	0 (0)	0 (0)	346 (100)	33 (9.5)	313 (90.5)	
13	Jiram et al. 2016 [40]	306		Cross-sectional	Peninsular					113 (36.9)	59 (52.2)	23 (20.4)	31 (52.5)
14	Othman et al. ^c^ [41]	94	2007–2010	Hospital	Borneo	94 (100)	93 (98.9)	0 (0)	1 (1.1)	94 (100)	92 (98.9)		2 (2.1)
15	Fornace et al. [23]	1147	2012–2014	Hospital	Borneo	1 (0.1)				206 (18)		20 (9.7)	
16	Lee et al. [27]	207	2012–2013	Hospital	Borneo					207 (100)	53 (25.7)	152 (73.4)	
17	Chua et al. [42]	229	2008–2010	Hospital	Borneo	215 (93.9)	214 (99.5)	0 (0)	1 (0.5)	226 (98.7)	185 (89.4)	36 (15.9)	2 (0.9)
18	Barber et al. [43]	774	2012–2013	Hospital	Borneo	774 (100)	757 (97.8)	0 (0)	17 (2.2)				
19	Vythilingam et al. [44]	4353	2009–2013	Hospital	Peninsular	1284 (29.5)	1262 (98.3)	0 (0)	22 (1.7)				
20	Foster et al. [45]	84	2010–2011	Hospital	Borneo					84 (100)	15 (17.9)	69 (82.1)	
21	Yusof et al. ^c^ [9]	457		Hospital	Borneo and Peninsular	457 (100)	274 (60)	181 (39.6)	2 (0.4)	453 (99.1)	185 (40.8)	256 (56.5)	12 (2.6)
22	Braima et al. [46]	1623	2006–2012	Hospital	Peninsular	1623 (100)	1522 (93.8)	75 (4.6)	26 (1.6)				
23	Goh et al. ^c^ [7]	189	2008–2011	Hospital	Borneo	189 (100)	186 (98.4)	0 (0)	3 (1.6)	178 (94.2)	134 (75.3)	42 (24)	2 (1.1)
24	Barber et al. ^c^ [47]	387	2010–2011	Hospital	Borneo	387 (100)	221 (57.1)	150 (38.8)	16 (4.1)	295 (100)	165 (55.9)	130 (44.1)	
25	Barber et al. ^c^ [48]	18,993	2009–2011	Hospital	Borneo	653 (3.4)	558 (85.5)	0 (0)	95 (14.5)	475 (97.9)	58 (12.2)	345 (72.3)	48 (10.1)
26	Khin et al. ^c^ [49]	445	2009	Hospital	Borneo	445 (100)	318 (71.5)	0 (0)	25 (5.6)	343 (100)		256 (74.6)	87 (25.4)
27	Norahmad et al. [50]	619	2008–2009	Hospital	Borneo	58 (9.4)	31 (53.4)						
28	William et al. ^c^ [51]	78	2007–2009	Hospital	Borneo	78 (100)	0 (0)	0 (0)	0 (0)	63 (100)	2 (3.2)	56 (88.9)	5 (7.9)
29	Barber et al. ^c^ [52]	220	2009	Hospital	Borneo	220 (100)	184 (83.6)	0 (0)	36 (16.4)	155 (96.3)	9 (5.8)	127 (81.9)	12 (7.7)
30	Daneshvar et al. [53]	188	2006–2008	Hospital	Borneo	188 (100)	60 (31.9)	121 (64.4)	7 (3.7)				
31	Lee et al. ^c^ [54]	47	1996	Hospital	Borneo	47 (100)	47 (100)	0 (0)	0 (0)	36 (76.6)	1 (2.8)	29 (80.6)	6 (16.7)
32	Gurpreet [55]	520	2000–2001	Cross-sectional	Peninsular	126 (24.2)	126 (100)	0 (0)	0 (0)				
33	Vythilingam et al. ^c^ [8]	111	2005–2008	Hospital	Borneo and Peninsular	111 (100)	108 (97.3)	0 (0)	3 (2.7)	111 (100)	33 (29.7)	65 (58.6)	13 (11.7)
34	Cox-Singh et al. ^c^ [56]	960	2001–2006	Hospital	Borneo	960 (100)	958 (99.8)	0 (0)	2 (0.2)	960 (100)	664 (69.2)	243 (25.3)	53 (5.5)
35	Nimir et al. [57]	382	1998–2003	Hospital	Peninsular	382 (100)	347 (90.8)	0 (0)	35 (9.2)				
36	Jamaiah et al. [58]	94	1999–2004	Hospital	Peninsular	94 (100)	89 (94.7)	0 (0)	5 (5.3)				
37	Jamaiah et al. [59]	86	1994–2003	Hospital	Peninsular	86 (100)	76 (88.4)	0 (0)	8 (9.3)				
38	Singh et al. ^c^ [60]	208	2000–2002	Hospital	Borneo	208 (100)	208 (100)	0 (0)	0 (0)	208 (100)	82 (39.4)	106 (51)	20 (9.6)
39	Koh et al. [61]	31	1996–2001	Hospital	Borneo	31 (100)	30 (96.8)	0 (0)	1 (3.2)				
40	Norhayati et al. [62]	310		Cross-sectional	Peninsular	34 (11)	34 (100)	0 (0)	0 (0)				
41	Singh et al. [63]	129		Cross-sectional	Borneo	36 (27.9)	31 (86.1)	0 (0)	5 (13.9)	43 (33.3)	32 (74.4)	0 (0)	11 (25.6)
42	Jamaiah et al. [64]	134	1983–1992	Hospital	Peninsular	134 (100)	123 (91.8)	0 (0)	11 (8.2)				
43	Singh et al. [65]	166		Hospital and Cross-sectional	Borneo	68 (41)	62 (91.2)	0 (0)	6 (8.8)	73 (44)	65 (89)	0 (0)	8 (11)
44	Sidhu et al. [66]	64	1984–1988	Hospital	Peninsular	64 (100)	62 (96.9)	0 (0)	2 (3.1)				
45	Gordon et al. [67]	268		Cross-sectional	Peninsular	60 (22.4)	50 (83.3)	0 (0)	10 (16.7)				
46	Lee et al. [68]	94	1986	Cross-sectional	Peninsular	45 (47.9)	23 (51.1)	0 (0)	22 (48.9)				

Pf = *P. falciparum*; Pv = *P.* *vivax*; Pm = *P.* *malariae*; Po = *P.* *ovale* and Pk = *P.* *knowlesi*

^a^Total of six studies has no data on period of sample collection. Some studies do not explicitly stratify the number of cases in each year

^b^Borneo including the states of Sabah and Sarawak in East Malaysia

^c^Studies with PCR diagnosis derived from a subset of microscopy positive data

In term of malaria diagnosis method, 28 studies utilized both microscopy and PCR, but in 18 of them, the samples tested for PCR were chosen from the microscopy positive cases for malaria species confirmation. Of those 18 studies tested for PCR, ten studies [7–9, 36–38, 41, 54, 56, 60] used all the microscopy positive cases, six studies [5, 33, 47, 49, 51, 52] used more than 70% of the cases, one study [48] used 2.5% randomly selected cases, and one study [39] used 19% of microscopy positive cases for either *P. malariae* or *P. knowlesi*. Furthermore, of the 28 studies, ten studies [12, 24, 25, 28, 32, 35, 42, 53, 63, 65] conducted PCR on all samples regardless of the microscopy results in order to trace the sub-microscopic infections. In addition, 18 studies utilized only one method of detection for malaria: 15 [34, 43, 44, 46, 50, 55, 57–59, 61, 62, 64, 66–68] by microscopy and three [27, 40, 45] by PCR.

Overall, the median sample sizes were 308 cases (range 31–18,993) for microscopy and 261 (range 47–4257) for PCR. Most microscopy diagnosis used Giemsa-stained thick and thin smears (n = 35), while the remaining studies (n = 8) used only thick smear. In studies using PCR methods (n = 31), the majority used conventional nested PCR (n = 22), followed by three studies used multiplex PCR and two studies used real-time PCR. Other studies (n = 4) reported combination of different PCR methods; conventional/multiplex/real-time PCR, loop-mediated isothermal amplification (LAMP) assays [38], conventional/multiplex PCR assays [28], conventional/real-time PCR assays [12], and multiplex/real-time PCR assays [27].

### Parasite and incidence rates by year of sampling

**Fig. 2:**
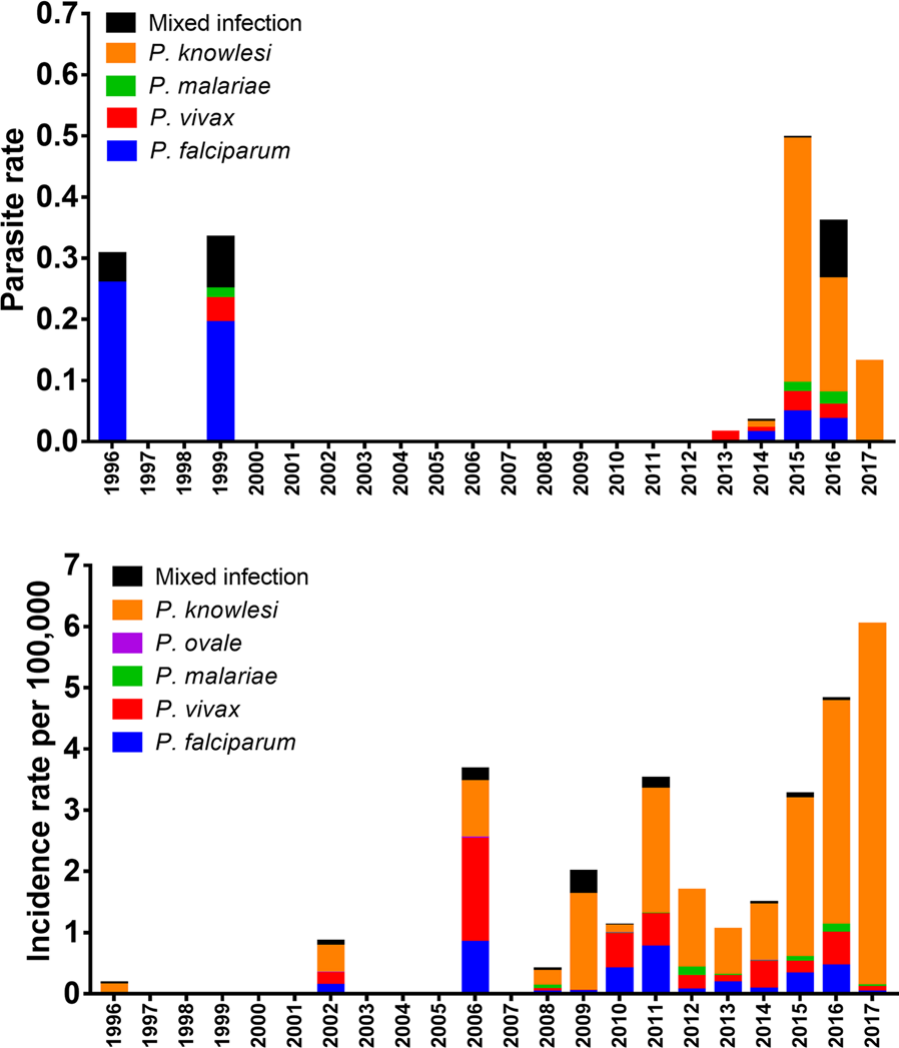
Parasite rate by year of sampling collection based of available PCR data from cross-sectional studies [24, 25, 35, 36, 40, 63, 65] between 1996 to 2017 (top). Incidence rate per 100,000 populations by year of sampling collection based on available PCR data from hospital-based studies [5, 7–9, 23, 27, 28, 33, 38, 39, 41, 42, 45, 47–49, 51, 52, 54, 56, 60] between 1996 to 2017 (bottom)

The parasite rate and average incidence rate by year of sampling are shown in Fig. 2. In total, seven cross-sectional [24, 25, 35, 36, 40, 63, 65] and 21 hospital-based studies [5, 7–9, 23, 27, 28, 33, 38, 39, 41, 42, 45, 47–49, 51, 52, 54, 56, 60] with available PCR data for malaria species (1996 to 2017) were used to calculate trends in parasite rate and incidence rate, respectively. Generally, over the 22-year period, species predominance shifted from *P. falciparum* before 2000 to *P. knowlesi* from 2015 onward. *Plasmodium knowlesi* parasite rate rose drastically from 0.003 in 2014 to 0.399 in 2015, but declined to 0.187 and 0.131 in 2016 to 2017, respectively. Similar trend for incidence rate was also observed across the 22 years. *Plasmodium falciparum* incidence decreased from 0.836 per 100,000 in 2006 to 0.016 per 100,000 in 2017, and *P. vivax* from 1.679 per 100,000 in 2006 to 0.080 per 100,000 in 2017. In contrast, *P. knowlesi* incidence rate rose steadily throughout the years from 0.029 per 100,000 in 1996 to 5.909 per 100,000 in 2017.

### Sensitivity and specificity of detection methods

**Fig. 3:**
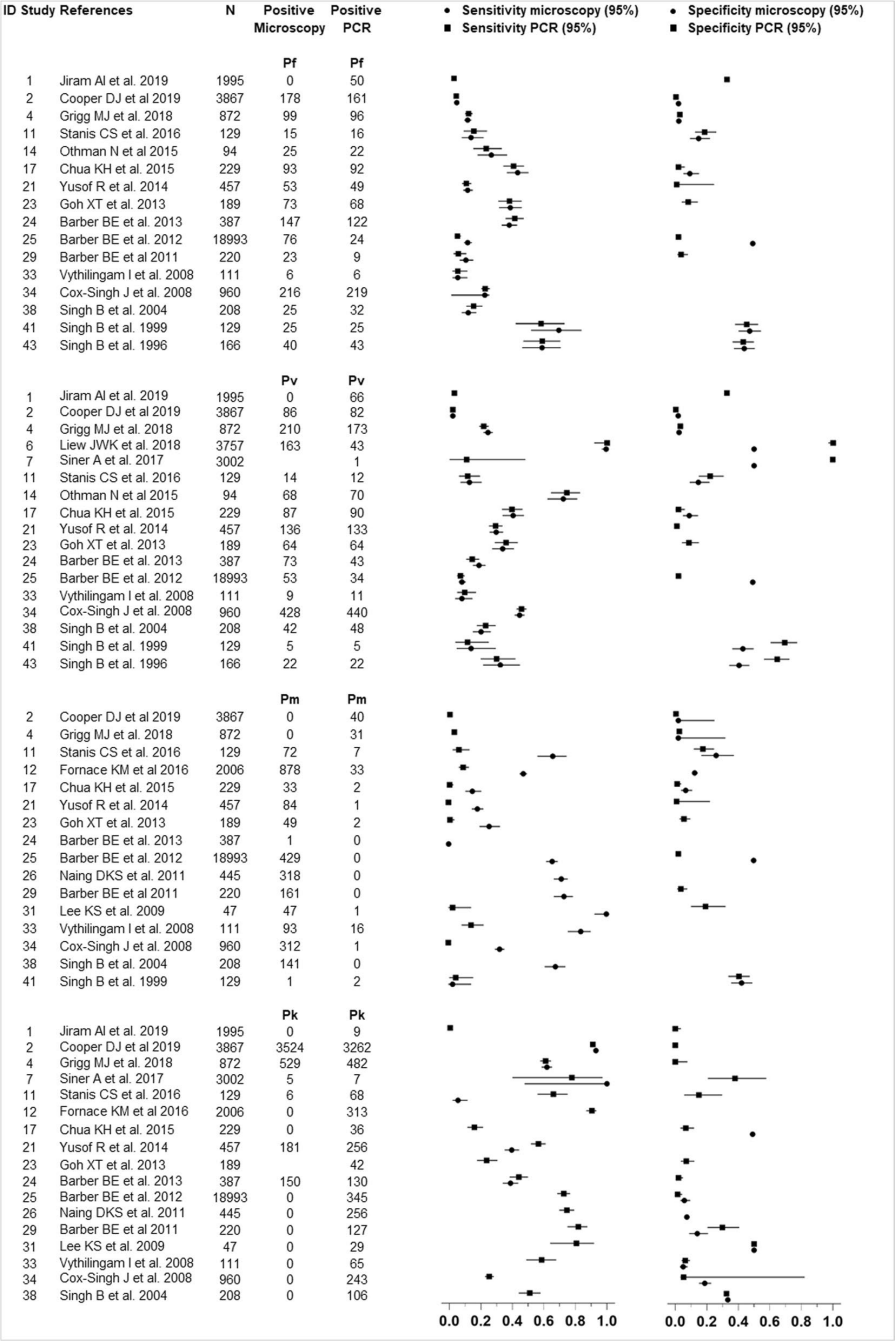
Performance of malaria diagnostic methods (microscopy and PCR) for detection of *Plasmodium falciparum*, *Plasmodium vivax*, *Plasmodium malariae* and *Plasmodium knowlesi* mono-infections

The performance of microscopy and PCR in detecting *Plasmodium* spp. are shown in Fig. 3. In total, 21 studies were included; 18 studies were undertaken in Malaysia Borneo [5, 7, 24, 25, 28, 33, 39, 41, 42, 47–49, 52, 54, 56, 60, 63, 65], one study was in Peninsular Malaysia [35] and two studies were in both Peninsular Malaysia and Malaysia Borneo [8, 9]. Overall, the summary estimate of sensitivity by microscopy was highest for *P. knowlesi* (35% [95% CI 34–36]), followed by *P. malariae* (25% [95% CI 24–26]) and *P. vivax* (14% [95% CI 14–15]), and lowest for *P. falciparum* (11% [95% CI 10–11]). Nevertheless, the summary estimate of specificity by microscopy was similar in all species ranged 40–43%.

Similar to microscopy, the summary estimate of sensitivity by PCR was highest for *P. knowlesi* with 56% (95% CI 55–57). Whereas, the summary estimate of sensitivity by PCR for *P. vivax, P. falciparum* and *P. malariae* were 14% (95% CI 13–15), 11% (95% CI 10–11) and 1.6% (95% CI 1.4–1.9), respectively. On the other hand, the specificities of summary estimate for species-specific were less than 35% with *P. knowlesi, P. vivax*, *P. falciparum* and *P. malariae* were 34.5% (95% CI 34–35), 34% (95% CI 33–35), 16.5% (95% CI 16–17) and 2.3% (95% CI 2–3), respectively.

## Discussion

Malaysia aims to eliminate malaria nationwide by 2020. Although the country has successfully eliminated indigenous transmission of all human malaria species [3], the incidence of malaria caused by *P. knowlesi* continues to infect a large number of people in remote parts of Malaysia [5, 11, 69]. This is the first systematic review to determine the pooled distribution of all malaria species and performance of major malaria diagnostic methods in Malaysia, based on English publications. The study has analysed 46 full-text publications reported since the 1980s in Malaysia.

In this study, malaria incidence caused by human malaria species indicates a decreasing trend from 2006 onwards. This finding is consistent with a recent study by Hussin et al. that showed the decreasing incidence of human malaria notified to the Ministry of Health, Malaysia between 2013 to 2017 [6]. This downward trend is a testament to the determination of the government and other parties to eliminate malaria in Malaysia. The Malaysia National Malaria Elimination Strategic Plan 2011–2020 has set the ultimate goal of stopping locally acquired malaria (except *P. knowlesi*) in the Peninsular region by 2015 and in the Malaysia Borneo region by 2020 [4, 17]. The plan outlines seven key actions for achieving the elimination goal including strengthening the malaria surveillance system through an online system, stepping up control activities through indoor residual spraying (IRS) and insecticide-treated net (ITN), ensuring early case investigation, prompt treatment and outbreak management, and enhancing community awareness and knowledge of malaria. All these efforts have resulted in significant reduction in overall malaria incidence in general over the last decade.

Over the past years, malaria species-specific analysis showed that *P. knowlesi* was the most dominant species, particularly in Malaysia Borneo i.e. East Malaysia. *Plasmodium knowlesi* cases rose steadily year by year and the incidence rate was highest at 5.909 per 100,000 in 2017. Other than the wide utilization of molecular diagnosis in health facilities, it has been hypothesized that the rise of knowlesi malaria cases in the country was associated with ecological changes, particularly by deforestation [11, 36, 70]. The expansion of deforestation may have disturbed the habitat of mosquito vectors and simian hosts, as well as enhanced contact with the humans. In addition, the loss of habitat along with malaria control practices may have contributed to a change in vector behaviour or vector shift, as has been seen in the Kinabatangan area in Sabah where the previously dominant malaria vector *Anopheles balabacensis* seems to have been replaced by *Anopheles donaldi* [11, 71]. This is supported by the spatial distribution of reported cases in Sabah which are clustered in forested areas [39]. Besides that, it has been indicated that male adults are at a higher risk of knowlesi malaria infection than females due to the formers’ occupational activity, which involves forestation or agricultural activities such as palm oil plantations that increase their exposure to the malaria vectors [72].

It was interesting to note that there was a dramatic reduction in both parasite and incidence rates of *P. vivax* and *P. falciparum* in Malaysia. *Plasmodium vivax* has been the main cause of human malaria in the country for the past 10 years and remains a health concern today [3]. In 2010, of the 5819 reported cases, approximately 60% were due to *P. vivax* [3]. Moreover, the potential for reactivation of dormant hypnozoites creates a number of difficulties for the elimination of malaria in the country [73]. The dramatic reduction of *P. vivax* observed in the present study also follows a steady increase in notification rate of *P. malariae*/*P. knowlesi*. In fact, over the past decade, a strong inverse correlation was observed between notification rates of *P. malariae*/*P. knowlesi* and *P. vivax* or *P. falciparum* [11, 72]. This may be caused by misdiagnosis by microscopy of true *P. falciparum* or *P. vivax* infections as *P. malariae*/*P. knowlesi* [10, 74]. Moreover, it is less common in the most misdiagnosed of true *P. knowlesi* as *P. falciparum* or *P. vivax* [75]. The effect of this finding, as would be expected with increasing incidence of *P. knowlesi* and reducing incidence of *P. vivax* and *P. falciparum*.

Widely, the detection of malaria parasites by light microscopy of Giemsa-stained blood films continues to be the gold standard for malaria diagnosis [26]. It is however imperfect, especially when it comes to differentiation of malaria species. In this review, 15 studies relied solely on microscopy for *Plasmodium* detection and species differentiation. The use of microscopy as the sole diagnostic method likely leads to an underestimation of the malaria burden in a specific population [76], particularly in *P. knowlesi* and *P. malariae* infections that usually present at densities below the limit for microscopic detection [24]. This review also provided insight in the disparity between microscopy and PCR in diagnosing malaria cases. Most of the prevalence by microscopy were on human malaria (62.4%), whereas the prevalence of zoonotic malaria (64.1%) were typically reported by PCR. Microscopically, *P. knowlesi* infection is commonly misdiagnosed as *P. malariae* infection and other *Plasmodium* infections (*P. falciparum* and *P. vivax*) due to their morphological similarities [9, 10]. Although microscopic diagnosis of *Plasmodium* species is known to be problematic, this study demonstrates that the increase in notifications is likely to represent a real increase in the incidence of *P. knowlesi*. In this regard, PCR tests play an important role in order to confirm that the infection is due to knowlesi malaria.

A number of caveats should be considered in this study. First, the data used in the included studies were not uniform. The data gathered was solely based on published articles readily available on the internet and did not include data from the ministry of health. Thus, the findings of this study might not reflect the totality of malaria situation in Malaysia. In addition, most studies focused on the distribution of *Plasmodium* based on clinical samples and not in the population. This is possibly due to logistical difficulties and financial costs to carry the surveys, with most studies conducted in the Malaysia Borneo, where access to household is limited by geographic barriers. Second, our search strategy could have missed potentially eligible studies, because identification of *Plasmodium* infections were often not the primary target of many epidemiological studies in Malaysia. Third, the small sample size in some studies did not allow the evaluation of possible source of a high variation between studies.

## Conclusion

This study outlined the epidemiological changes in *Plasmodium* species distribution in Malaysia. Malaria cases shifted from predominantly human malaria especially *P. falciparum* and *P. vivax* to *P. knowlesi* in the early 2000s. *Plasmodium knowlesi* is now responsible for the majority of malaria cases in the country. Therefore, malaria case notification and interventions in Malaysia should be strengthened and reinforced to achieving the final goal of malaria elimination in the country.

## Data Availability

The datasets used and/or analysed during the current study are available from the corresponding author on reasonable request.

## Abbreviations

IRS: Indoor residual spraying
ITN: Insecticide-treated net
LAMP: Loop-mediated isothermal amplification
PCR: Polymerase chain reaction
RDT: Rapid diagnostic test
WHO: World Health Organization
